# The VEGF expression associated with prognosis in patients with intrahepatic cholangiocarcinoma: a systematic review and meta-analysis

**DOI:** 10.1186/s12957-022-02511-7

**Published:** 2022-02-21

**Authors:** Chunping Cai, Xiaoji Wang, Qiurong Fu, Ai Chen

**Affiliations:** 1 Department of Liver and Gallbladder Surgery, The First Affiliated Hospital of Hainan Medical University, Haikou, 570102 Hainan China; 2 Department of Nursing, The First Affiliated Hospital of Hainan Medical University, Haikou, Hainan China

**Keywords:** VEGF, Intrahepatic cholangiocarcinoma, Prognosis, Meta-analysis

## Abstract

**Objective:**

To systematically evaluate the relationship between vascular endothelial growth factor (VEGF) and prognosis of intrahepatic cholangiocarcinoma by meta-analysis.

**Methods:**

We systematically searched relevant studies in the databases of PubMed, Embase, Cochrane Library, CNKI, Wangfang, and Web of Science, with search dates limited to September 1, 2021. We extracted relevant data, including prognosis and clinicopathological features of patients with different expressions of VEGF in intrahepatic cholangiocarcinoma. The combined hazard ratio (HR), odds ratio (OR), and 95% confidence interval (CI) were calculated to evaluate the link strength between VEGF and prognosis of cholangiocarcinoma patients.

**Results:**

A total of 7 eligible studies with 495 patients were included in this meta-analysis. The results showed that the high expression of VEGF was significantly related to poor overall survival (OS) (HR = 1.93, 95% CI 1.52–2.46, *P* < 0.05) in patients with intrahepatic cholangiocarcinoma. Moreover, high expression of VEGF in tumor tissues associated with lymph node metastasis (LNM) (OR = 6.79, 95% CI 3.93–11.73, *P* < 0.05) and advanced TNM stage (OR = 4.35, 95% CI 2.34–8.07, *P* < 0.05) in intrahepatic cholangiocarcinoma. Sensitivity analysis shows that the meta-analysis results are stable and reliable.

**Conclusion:**

The expression of VEGF is related to the OS of patients with intrahepatic cholangiocarcinoma, and the OS of patients with high expression of VEGF is shorter. VEGF may be a novel predictor of intrahepatic cholangiocarcinoma patients.

**Trial registration:**

PROSPERO (CRD42022297443).

**Supplementary Information:**

The online version contains supplementary material available at 10.1186/s12957-022-02511-7.

## Introduction

Intrahepatic cholangiocarcinoma is a common malignant tumor in clinical practice. Early diagnosis is not easy because of the complicated anatomical relationship of tissue and the lack of specific tumor markers [1]. With the progression of the disease, longitudinal skip metastasis and lateral infiltration metastasis are easy to occur, which has serious adverse effects on the prognosis [2]. Therefore, it is of great significance to explore the pathological factors affecting the invasion and metastasis of intrahepatic cholangiocarcinoma.

Vascular endothelial growth factor (VEGF), with a relative molecular weight of 34,000~45,000, is a glycosylated secretory peptide factor isolated and purified from the bovine follicular stellate cells culture medium by Ferrara in 1989 [3]. The VEGF family includes five secretory glycoproteins, including VEGF-A, VEGF-B, VEGF-C, VEGF-D, and placental factors [4]. Members of the VEGF family activate the angiogenesis signal pathway by corresponding binding receptors and regulating physiological and pathological environments related to proliferation, differentiation, and vascular migration of vascular endothelial cells [5]. The combination of VEGF-A and VEGFR1 can promote the mesodermal differentiation to form vascular endothelial cells and maintain the integrity of vascular endothelium and the order of vascular morphology [6]. Previous studies have shown that VEGF is closely related to the occurrence and development of various tumors, and its expression is significantly related to the pathological grade, clinical stage, and lymph node metastasis of malignant tumors such as lung cancer, prostate cancer, stomach cancer, breast cancer, and colorectal cancer [7]. Bevacizumab is a recombinant humanized monoclonal antibody, containing the structural region of a human antibody, which can selectively bind to VEGF and block its biological activity, thus blocking VEGF-mediated tumor angiogenesis and delaying tumor growth [8]. It is now widely used to treat advanced malignant tumors in combination with radiotherapy and chemotherapy [9]. Ramucirumab is mainly used for the treatment of gastric cancer, lung cancer, colorectal cancer, and other malignant tumors. A phase III clinical trial found that ramucirumab combined with docetaxel has a significant advantage in the progression-free survival time of patients with locally advanced or metastatic urothelial cancer after failure of platinum-based chemotherapy [10, 11].

Although previous studies have analyzed the correlation mechanism between VEGF and prognosis in cholangiocarcinoma, the relationship between VEGF and prognosis and their effect on survival status has not been reported in intrahepatic cholangiocarcinoma. Therefore, this study attempted to analyze the relationship between the expression of VEGF in intrahepatic cholangiocarcinoma and its clinical significance by meta-analysis.

## Methods

### Search strategy

We prospectively registered this systematic review and meta-analysis with PROSPERO (CRD42022297443) and followed the Preferred Reporting Items for Systematic Reviews and Meta-Analysis (PRISMA) guidelines for this research. The search was conducted in PubMed, Embase, Cochrane Library, CNKI, China Wangfang, and Web of Science to retrieve the published literature on the relationship between VEGF and prognosis of intrahepatic cholangiocarcinoma up to September 1, 2021. The search terms were “VEGF,” “intrahepatic cholangiocarcinoma,” “survival,” “prognosis,” and “recurrence”; combined theme word, MeSH terms, and comprehensive keyword retrieval were conducted respectively according to the characteristics of different databases. The complete search strategy in PubMed was shown in Additional file 1: Table S1. The two researchers searched each database independently and finally cross-checked. Any conflict of terms was resolved through group discussions. There were no restrictions on language.

### Inclusion and exclusion criteria

This study aims to analyze the role of VEGF in intrahepatic cholangiocarcinoma participants, and the inclusion criteria were as follows: (a) articles that explored the association between VEGF expression and cancer prognosis; (b) studies with participants divided into high and low VEGF expression groups; (c) articles that described related clinicopathologic parameters such as age, gender, LNM, TNM stage, and tumor size; (d) the inclusion of sufficient data for the computation of hazard ratio (HR) and corresponding 95% confidence intervals (CI); and (e) articles that the expression of VEGF was detected by PCR.

Exclusion criteria were as follows: (a) duplicate publications; (b) reviews, letters, case reports, and nonhuman subject research; and (c) articles without usable data.

### Data extraction and quality assessment of primary studies

Data extraction and study quality assessment were independently completed by two researchers. Literature was screened according to inclusion and exclusion criteria, and relevant data were extracted. The basic research information to collect was as follows: author, country and publication time, sample size, cutoff value, and original data (hazards ratio and 95% confidence interval). If only Kaplan-Meier survival curve data were available for eligible studies, data such as HR and 95% CI were extracted from the graphs using the Engauge Digitizer (version 4.1) software [12]. If there was disagreement, two researchers would discuss it together. If necessary, a third researcher would be invited to participate in the discussion, and a unified result would be reached. The Newcastle-Ottawa Scale was mainly used to evaluate case-control studies. The quality of the included literature was evaluated according to the literature quality evaluation table (Newcastle-Ottawa Scale) [13]. NOS has an overall score of 9. The study with a score of ≥ 6 was considered high quality.

### Statistical analysis

Meta-analysis was performed using the Review Manager 5.3 software (the Cochrane Collaboration, Copenhagen, Denmark) and commercial software programs (STATA, version 12.0; College Station, TX, USA). Heterogeneity among studies was judged according to the size of statistic *I*^2^. When *I*^2^ was > 50%, there was heterogeneity among studies, and a random-effects model was used. Otherwise, a fixed-effects model was adopted. The combined effect size was HR and its 95% confidence interval (CI), and the statistical significance of HR value was analyzed by the *Z*-test. Pooled odds ratio (OR) and 95% CI were used to evaluate the relationship between clinicopathological features and VEGF. *P* < 0.05 was considered statistically significant. Sensitivity analysis was used to evaluate the stability of the findings, and funnel plots were used to detect the presence of publication bias.

## Results

### Characteristics of eligible studies

**Fig. 1:**
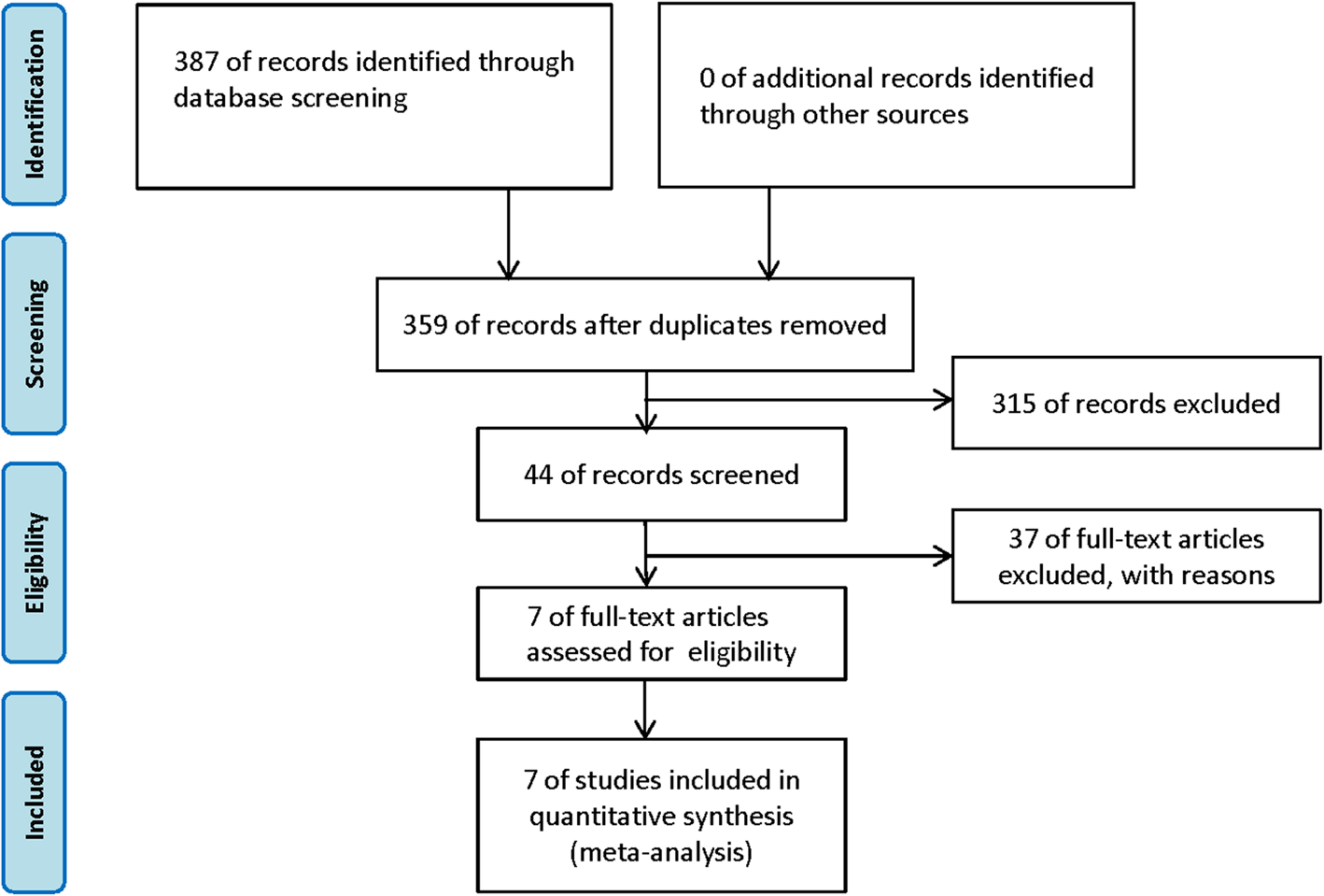
The flow diagram of this meta-analysis

A flow diagram of the literature screen and selection was performed for this study (Fig. 1). According to the search strategy, a total of 387 studies were retrieved, and 315 studies were excluded from repeated publications, reviews, abstracts, case reports, and non-prognostic and nonmalignant tumor-related studies. After reading the full text, 37 studies without survival outcome-related indicators were excluded, and 7 studies were finally included [14–20]. NOS scores range from a minimum of 6 to a minimum of 8 (Additional file 2: Table S2). Quantitative reverse transcription-polymerase chain reaction (qRT-PCR) was used for detection (Table 1).

**Table 1:** Characteristics of the included studies in this meta-analysis

Study	Year	Country	Cancer type	Total	Tumor stage	Method	Cutoff	VEGF expression				Survival analysis	Multivariate analysis	HR statistic	HR (95% CI)	Follow-up months	NOS score
								High expression	High with LNM	Low expression	Low with LNM						
Byung [14]	2006	Korea	ICC	79	I–IV	RT-qPCR	Median	36	7	21	3	OS	Rep	SC	2.02 (1.18–3.46)	72	8
Liu [15]	2010	China	ICC	86	I–IV	RT-qPCR	Median	69	27	11	1	OS	NR	SC	1.95 (0.73–5.16)	140	7
Shinichi [16]	2008	Japan	ICC	62	I–IV	RT-qPCR	Mean	88	19	62	19	OS	Rep	SC	1.74 (1.07–2.82)	120	7
Wang [17]	2009	China	ICC	130	I–IV	RT-qPCR	Median	69	27	11	1	OS	NR	SC	2.85 (1.08–7.56)	140	8
Xiao [18]	2012	China	ICC	60	I–IV	RT-qPCR	Mean	47	19	11	2	OS	NR	SC	1.66(0.71–3.90)	60	7
Xu [19]	2015	China	ICC	435	I–IV	RT-qPCR	Mean	65	13	27	2	OS	Rep	SC	3.003 (1.016–8.875)	100	8
Zhu [20]	2020	China	ICC	102	I–IV	RT-qPCR	Median	122	73	21	5	OS	NR	SC	1.82 (1.17–2.82)	60	7

*ICC* intrahepatic cholangiocarcinoma, *HR* hazard ratio, *LNM* lymph node metastasis, *NR* no report, *OS* overall survival, *Rep* report, *RT-qPCR* real-time quantitative polymerase chain reaction, *SC* survival curve, *NOS* Newcastle-Ottawa Scale

### Association between VEGF and prognosis of intrahepatic cholangiocarcinoma patients

**Fig. 2:**
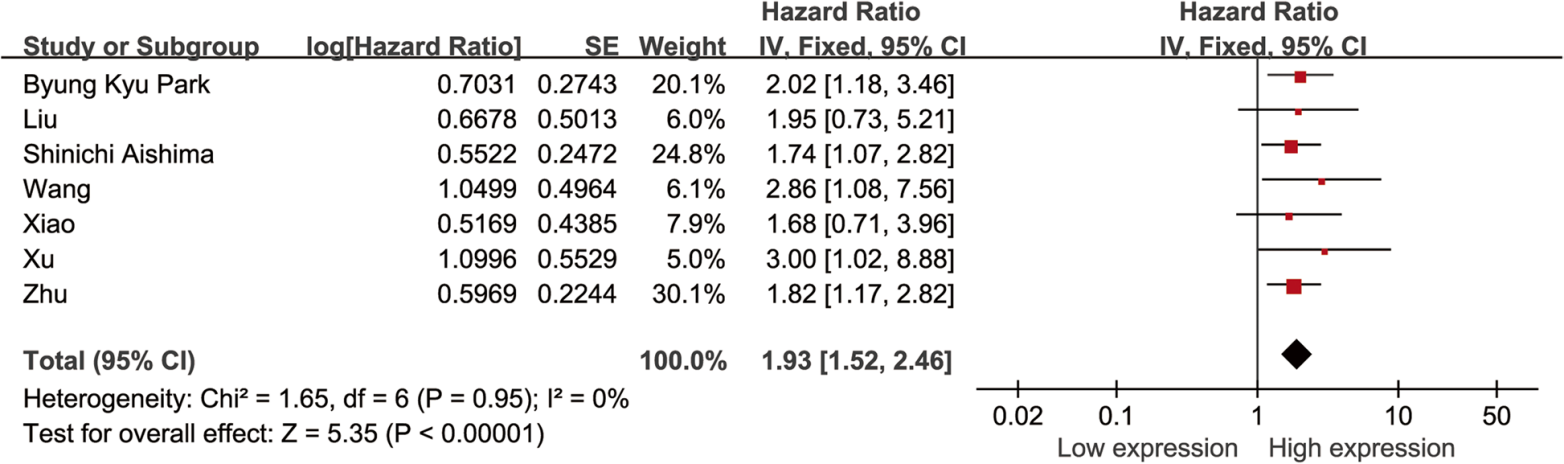
Meta-analysis of the pooled HR and OS in intrahepatic cholangiocarcinoma with the expression level of VEGF. OS, overall survival; HR, hazard ratio; VEGF, vascular endothelial growth factor

A total of 7 studies on the relationship between VEGF expression and overall survival (OS) in patients with intrahepatic cholangiocarcinoma met the inclusion criteria, including a total of 495 patients with OS as research endpoint. Combined HR and 95% CI were collected from 7 studies. By applying the fixed-effects model (*I*^2^ = 0%, *P* = 0.95), the result indicated that high expression level of VEGF correlated with poor OS in patients with intrahepatic cholangiocarcinoma (pooled HR = 1.93, 95% CI 1.52–2.46; *P* < 0.001) (Fig. 2). The results showed that the expression of VEGF was related to the OS of patients with intrahepatic cholangiocarcinoma, implying that high expression of VEGF was an unfavorable factor affecting the prognosis of patients with intrahepatic cholangiocarcinoma.

### Subgroup analysis

**Fig. 3:**
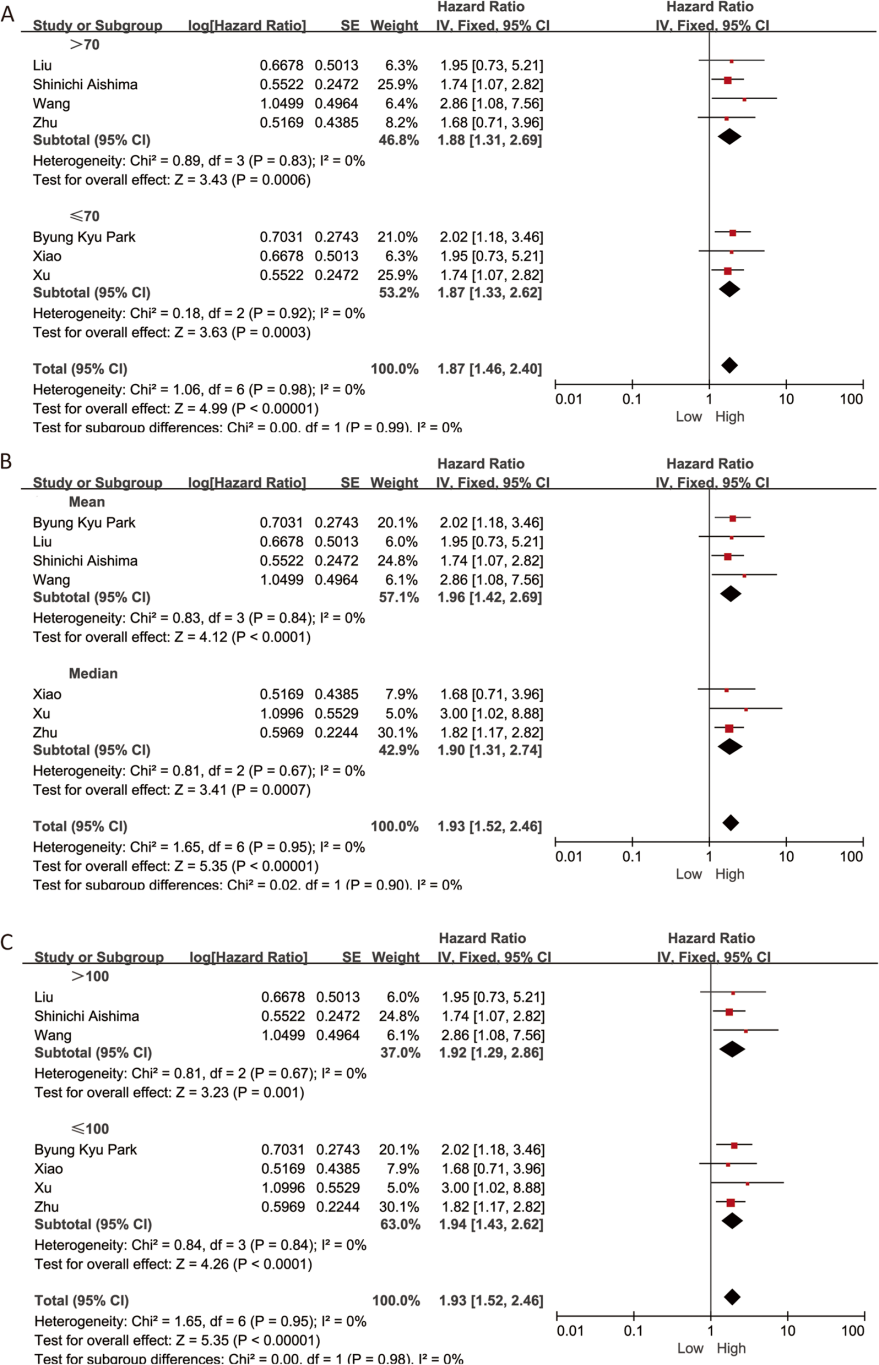
Subgroup analysis of HR for the association between VEGF expression and overall survival. **A** Sample size. **B** follow-up time. **C** cutoff value. VEGF, vascular endothelial growth factor; HR, hazard ratio

These results confirmed that high VEGF expression in cancer tissues is a significant biomarker for the poor prognosis of patients with intrahepatic cholangiocarcinoma. Although the heterogeneity is not significant, we also performed a subgroup analysis stratified for cutoff value, sample size, and follow-up time. After stratification by sample size, we observed that VEGF was a prognostic factor in groups with sample size ≤ 70 patients (HR = 1.87, 95% CI 1.46–2.40, *P* < 0.001) and sample size > 70 patients (HR = 1.88, 95% CI 1.31–2.69, *P* < 0.001) (Fig. 3A). According to the cutoff value of distinguishing patients with high expression and low expression, the studies were divided into the median or mean groups. For the cutoff value, we found that the predictive value was significant both in the median group and mean group (HR = 1.93, 95% CI 1.52–2.46, *P* < 0.001) (Fig. 3B). Subsequently, we found that VEGF could act as a prognostic factor in groups with follow-up time ≥ 100 mouths and < 100 mouths (HR = 1.93, 95% CI 1.52–2.46, *P* < 0.001) with low heterogeneity (*I*^2^ = 0.0%, *P* > 0.1) (Fig. 3C).

### Risk of bias and sensitivity analysis

**Fig. 4:**
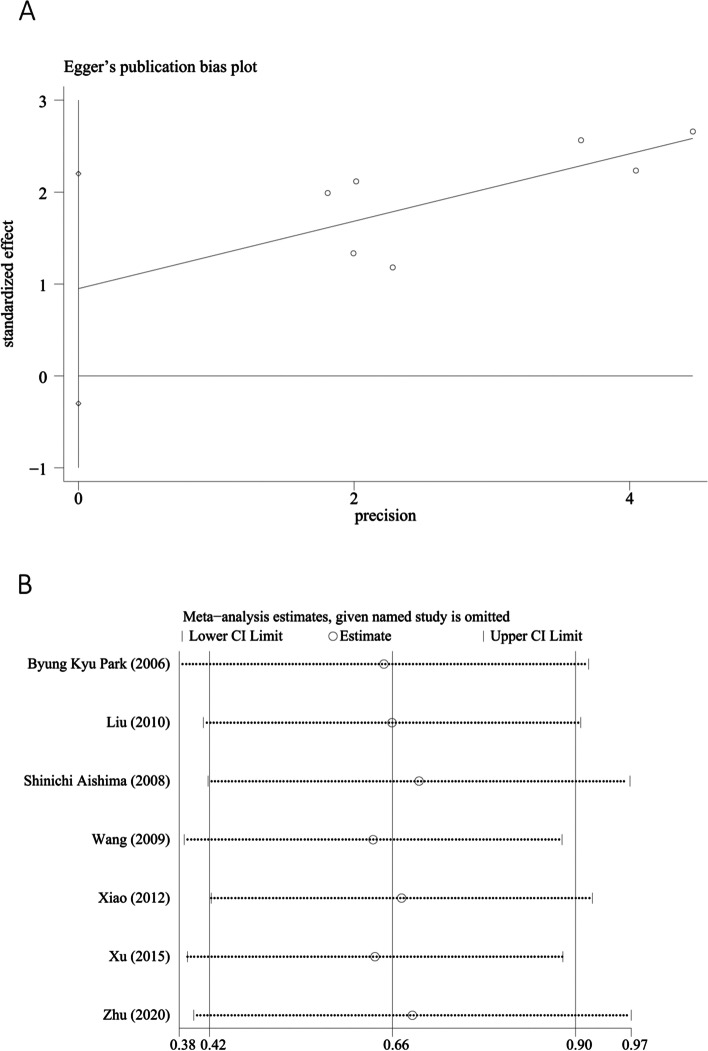
Detection of publication bias and sensitivity analysis of the meta-analysis. **A** Egger’s funnel plot. **B** sensitivity analysis of poor HR for heterogeneity analysis. HR, hazard ratio

The Egger’s funnel plot (Fig. 4A) indicated no significant publication bias (Z = 1.50; *P* = 0.13) (*P* > 0.05). For the studies on the relationship between VEGF expression and OS in patients with intrahepatic cholangiocarcinoma, there were no significant changes in pooled HR when each study was removed, indicating the stability and reliability of results (Fig. 4B).

### Association between VEGF and clinicopathological features in intrahepatic cholangiocarcinoma patients

**Fig. 5:**
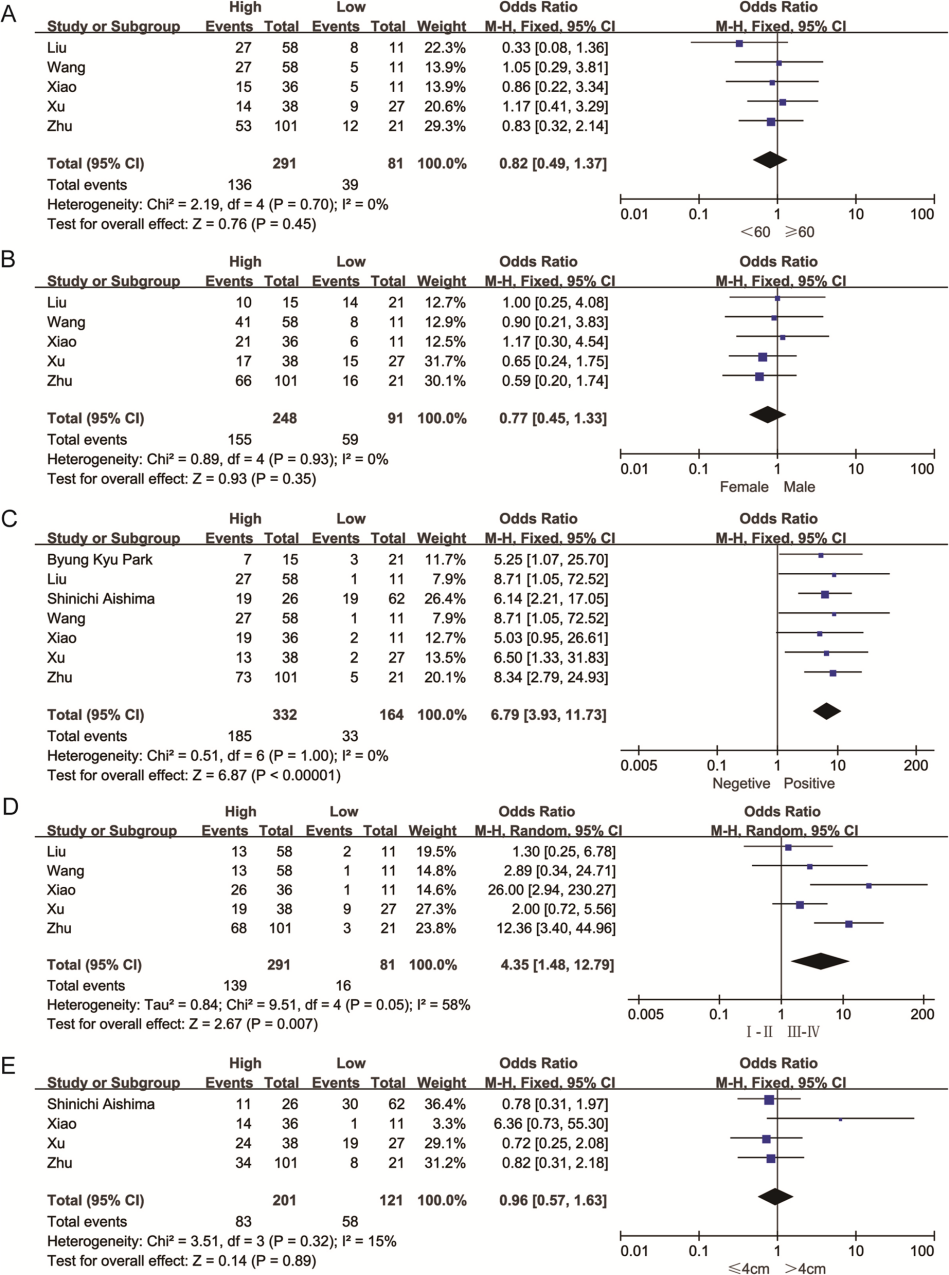
Meta-analysis of the clinicopathologic features in intrahepatic cholangiocarcinoma with the expression level of VEGF. **A** Age (> 50 vs. ≤ 50). **B** gender. **C** LNM (yes vs. no). **D** TNM stage (III–IV vs. I–II). **E** tumor size (> 5 cm vs. ≤ 5 cm)

To analyze the association between VEGF and clinicopathological characteristics in intrahepatic cholangiocarcinoma patients, we conducted the pooled results including age in five studies, gender in five studies, lymph node metastasis in six studies, TNM stage in five studies, and tumor size in four studies with total patients of 496. The results (Table 2) indicated that no significant association was detected between VEGF expression and age (OR = 0.82, 95% CI 0.49–1.37, *P* = 0.45), gender (OR = 0.77, 95% CI 0.45–1.33, *P* = 0.35) (Fig. 5A, B), and tumor size (OR = 0.96, 95% CI 0.57–1.63, *P* = 0.89) (Fig. 5E). Remarkably, high VEGF expression was significantly correlated with LNM (OR = 6.79, 95% CI 3.93–11.73, *P* < 0.001) and advanced TNM stage (OR = 4.35, 95% CI 1.48–12.79, *P* < 0.001) with heterogeneity (*I*^2^ = 58%, *P* = 0.05) (Fig. 5C, D).

**Table 2:** Main results of the association between VEGF and characteristics of patients with cholangiocarcinoma

Stratified analysis	No. of studies	No. of patients	Pooled HR/OR (95% CI)	***p***-value	Heterogeneity		
					***I***^**2**^, %	***p***-value	Model
OS							
Overall	7	496	1.93 (1.52, 2.46)	< 0.001	0	0.95	FEM
Clinicopathological features							
Age (> 60 vs. ≤ 60)	5	372	0.82 (0.49, 1.37)	0.45	0	0.70	FEM
Gender (male vs. female)	5	339	0.77 (0.45, 1.33)	0.35	0	0.93	FEM
LNM (yes vs. no)	7	496	6.79 (3.93, 11.73)	< 0.001	0	1.00	FEM
TNM stage (III–IV vs. I–II)	5	372	4.35 (1.48, 12.79)	0.007	58	0.05	REM
Tumor size (> 5 cm vs. ≤ 5 cm)	4	322	0.96 (0.57, 1.63)	0.89	15	0.32	FEM

*CI* confidence interval, *HR* hazard ratio, *OR* odds ratio, *LNM* lymph node metastasis, *OS* overall survival, *TNM* tumor node metastasis, *vs* versus, *FEM* fixed-effects model, *REM* random-effects model

## Discussion

Malignant tumor poses a serious threat to human health [21]. Previous studies have proved that VEGF plays an essential role in the occurrence and development of tumors, but the current research results are inconsistent [16, 19, 22]. In this study, a meta-analysis was conducted on the relationship between VEGF and the prognosis of patients with intrahepatic cholangiocarcinoma.

The results showed that VEGF expression was associated with the OS of patients with intrahepatic cholangiocarcinoma, and the OS of patients with high expression of VEGF was shorter. The results revealed that the *I*^2^ value of subgroup analysis based on literature quality was low, suggesting no significant heterogeneity among the included studies. At the same time, we investigated the relationship between VEGF expression level and LNM and advanced TNM stage. The results showed that patients with high expression of VEGF in cancer tissues were more likely to develop lymph node metastasis and tumor progression. In funnel plot analysis of pooled OR, it is found that the funnel plot is asymmetrical, which may be related to the small sample test. Cholangiocarcinoma is a malignant tumor occurring from the extrahepatic biliary tract to the hepatic hilus and the lower end of the bile duct [23]. According to relevant statistics, it is more common in the group of 50~70 years old [24]. The incidence presents a gradually increasing trend with low 5-year survival rate, so the prevention and treatment situation are grim [25]. Therefore, it is necessary to explore the changes of related factors to find new therapeutic approaches to strengthen the efficacy and prolong the survival time.

VEGF, a kind of vascular endothelial growth factor, plays an essential role in the angiogenesis of various malignant tumors and is closely related to the occurrence and development of tumors [26]. VEGF can accelerate the invasion and metastasis of cells and promote the deterioration of the disease by participating in angiogenesis [27]. It has been reported that VEGF participates in the invasion and metastasis of cervical cancer by binding specific receptors and inducing lymphatic endothelial cells and angiogenesis [27]. On this basis, this study found that VEGF was highly expressed in intrahepatic cholangiocarcinoma, which had an essential influence on the invasion and metastasis of the tumor.

Previous research reported that VEGF could bind and activate vascular endothelial growth factor receptor (VEGFR) via the phosphatidylinositol 3-kinase (P13K)/protein kinase B (Akt) and mitogen extracellular kinase (MEK)/extracellular signal-regulated kinase (ERK) signaling pathways [28]. It also stimulates and accelerates the mitosis and proliferation of lymphatic endothelial cells, thus triggering lymph node metastasis [29]. In addition, the overexpression of VEGF-C can increase the diameter of peripheral lymphatic vessels and increase the chance of tumor cells invading the lymphatic system to promote tumor metastasis [30]. On the other hand, VEGF-C can promote the paracrine or autocrine of chemokines and mitosis factors of lymphatic endothelial cells. Previous study reported that VEGF and VEGFR2 are highly expressed in cytotoxic T lymphocytes, and VEGF might promote the occurrence and development of tumors by inhibiting immune response [31]. With the advancement of precision medicine, the combined application of anti-vascular therapy with other targeted therapies and immunotherapy provides more survival time for patients with malignant tumors [32].

Our study indicated that VEGF is closely correlated in bile duct carcinoma tissues and is significantly correlated with arteriovenous invasion, lymph node metastasis, and clinical stage. Combined detection is helpful to provide a reference for clinical evaluation of disease and prediction of survival status.

However, there are several limitations that should be pointed out in this study. First, there were only 7 studies in this meta-analysis with moderate sample capacity. Second, postoperative chemotherapy and radiotherapy would affect the overall survival of the subjects and bring heterogeneity to this study. Inclusion of patients receiving adjuvant therapy in this meta-analysis needs to be further clarified. Third, in some studies, the way of estimating HR and 95% CI from the Kaplan-Meier curve might affect the accordance of the result. Fourth, the ways of distinguishing the cutoff value of VEGF expression groups may cause heterogeneity. Fifth, due to the limitations of sample size and detection methods, the synergistic mechanism of VEGF in intrahepatic cholangiocarcinoma still needs to be further clarified. To affirm its prognostic significance, more studies with larger sample sizes and other ethnic groups are required to perform.

In conclusion, high expression of VEGF was significantly related to a worse prognosis in intrahepatic cholangiocarcinoma. Our results indicated that VEGF could serve as a molecular biomarker to predict the prognosis of intrahepatic cholangiocarcinoma.

## Supplementary Information

**Additional file 1:** **Table S1.** Search strategy in Pubmed.

**Additional file 2:** **Table S2.** Study quality was assessed according to the Newcastle-Ottawa Scale.

## Data Availability

The datasets used and/or analyzed during the current study are available from the corresponding author on reasonable request.

## Abbreviations

VEGE: Vascular endothelial growth factor
HR: Hazard ratio
OR: Odds ratio
95% CI: 95% confidence interval
OS: Overall survival
LNM: Lymph node metastasis
CNKI: Chinese National Knowledge Infrastructure
VEGFR: Vascular endothelial growth factor receptor
qRT-PCR: Quantitative real-time polymerase chain reaction
SC: Survival curve
NOS: Newcastle-Ottawa Scale
