# Supplementary material for: The VEGF expression associated with prognosis in patients with intrahepatic cholangiocarcinoma: a systematic review and meta-analysis
Source: World J Surg Oncol. 2022 Feb 21;20:40. doi: 10.1186/s12957-022-02511-7 (PMC8859901; doi:10.1186/s12957-022-02511-7)
Supplement: Supplementary file 1 — Additional file 1: Table S1. Search strategy in Pubmed. [file 12957_2022_2511_MOESM1_ESM.docx]

Supplementary table 1. Search strategy in Pubmed.

| Queries in PubMed before Sep 1, 2021 | | |
| --- | --- | --- |
| Search | Query | Items  found |
| #1 | ("vascular endothelial growth factor"[Title/Abstract]) OR (VEGF[Title/Abstract]) OR (VEGF[MeSH Terms])) | 98991 |
| #2 | (("intrahepatic cholangiocarcinoma"[Title/Abstract]) OR (ICC[Title/Abstract])) OR ("liver tumor"[Title/Abstract]) | 34078 |
| #3 | ((("lymph node metastasis"[Title/Abstract]) OR (survival[Title/Abstract])) OR (prognosis[Title/Abstract])) OR (recurrence[Title/Abstract]) | 1569210 |
| #4 | #1 AND #2 AND #3 | 54 |
