# Supplementary material for: The VEGF expression associated with prognosis in patients with intrahepatic cholangiocarcinoma: a systematic review and meta-analysis
Source: World J Surg Oncol. 2022 Feb 21;20:40. doi: 10.1186/s12957-022-02511-7 (PMC8859901; doi:10.1186/s12957-022-02511-7)
Supplement: Supplementary file 2 — Additional file 2: Table S2. Study quality was assessed according to the Newcastle-Ottawa Scale. [file 12957_2022_2511_MOESM2_ESM.docx]

**Supplementary table 2.** Study quality was assessed according to the Newcastle-Ottawa Scale

| **study** | **Selection** | | | | **comparability** | | **outcome** | | | total |
| --- | --- | --- | --- | --- | --- | --- | --- | --- | --- | --- |
|  | Adequacy of case definition | Representativeness of the cases | Selection of Controls | Definition of Controls | Ascertainment of exposure | Ascertainment  of detection  method | Ascertainment  of cut-off | Assessment  of outcome | Adequate  follow up |  |
| Byung ^[14]^ | 1 | 1 | 1 | 1 | 1 | 0 | 1 | 1 | 1 | 8 |
| Liu ^[15]^ | 1 | 1 | 1 | 0 | 0 | 1 | 1 | 1 | 1 | 7 |
| Shinichi ^[16]^ | 1 | 1 | 1 | 0 | 1 | 1 | 1 | 1 | 0 | 7 |
| Wang ^[17]^ | 1 | 1 | 1 | 1 | 1 | 1 | 1 | 1 | 0 | 8 |
| Xiao ^[18]^ | 1 | 1 | 1 | 0 | 1 | 1 | 0 | 1 | 1 | 7 |
| Xu ^[19]^ | 1 | 1 | 1 | 1 | 1 | 1 | 1 | 1 | 0 | 8 |
| Zhu ^[20]^ | 1 | 1 | 0 | 1 | 0 | 1 | 1 | 1 | 1 | 7 |
